# Global molecular alterations involving recurrence or progression of pediatric brain tumors

**DOI:** 10.1016/j.neo.2021.11.014

**Published:** 2021-12-03

**Authors:** Fengju Chen, Darshan S. Chandrashekar, Michael E. Scheurer, Sooryanarayana Varambally, Chad J. Creighton

**Affiliations:** a Dan L. Duncan Comprehensive Cancer Center Division of Biostatistics, Baylor College of Medicine, Houston, TX, 77030, USA; b O'Neal Comprehensive Cancer Center, University of Alabama at Birmingham, Birmingham, AL, 35233, USA; c Molecular and Cellular Pathology, Department of Pathology, University of Alabama at Birmingham, Birmingham, AL, 35233, USA; d Department of Pediatrics, Baylor College of Medicine, Houston, TX, 77030, USA; e Texas Children's Cancer Center, Texas Children's Hospital, Houston, TX, 77030, USA; f The Informatics Institute, University of Alabama at Birmingham, Birmingham, AL, 35233, USA; g Human Genome Sequencing Center, Baylor College of Medicine, Houston, TX, 77030, USA; h Department of Medicine, Baylor College of Medicine, Houston, TX, 77030, USA

**Keywords:** CBTTC, The Children's Brain Tumor Tissue Consortium, CBTN, Children's Brain Tumor Network, CPTAC, Clinical Proteomic Tumor Analysis Consortium, TCGA, The Cancer Genome Atlas, GSEA, Gene Set Enrichment Analysis, Recurrence, Progression, Pediatric brain tumors, RNA-sequencing, Proteomics

## Abstract

**Background:**

We aimed to identify molecular changes in recurrent or progressive pediatric brain tumors, as compared to the corresponding initial tumors from the same patients, using genomic, transcriptomic, and proteomic data from a unique and large cohort of 55 patients and 63 recurrent or progressive tumors from the Children's Brain Tumor Tissue Consortium, representing various histologic types.

**Methods:**

We carried out paired analyses for each gene between recurrent/progressive and initial tumor groups, using RNA-sequencing and mass spectrometry-based proteomic data. By whole-genome sequencing (WGS) analysis, we also examined somatic DNA events for a set of cancer-associated genes.

**Results:**

Of 44 patients examined by WGS, 35 involved at least one cancer-associated gene with a somatic alteration event in a recurrent or progressive tumor that was not present in the initial tumor, including genes *NF1, CDKN2A, CCND2, EGFR*, and *MYCN*. By paired analysis, 68 mRNA transcripts were differentially expressed in recurrent/progressive tumors with p<0.001, and these genes could predict patient outcomes in an independent set of pediatric brain tumors. Gene transcript-level associations with recurrence or progression were enriched for protein-level associations. There was a significant overlap in results from pediatric brain tumors and results from adult brain tumors from The Cancer Genome Atlas. Unsupervised analysis defined five subsets of recurrent or progressive tumors, with differences in gene expression and overall patient survival.

**Conclusions:**

Our study uncovers genes showing consistent expression differences in recurrent or progressive tumors. These genes may provide molecular clues as to processes or pathways underlying more aggressive pediatric brain tumors.

## Introduction

Advances in surgical and adjuvant therapy have improved the survival rates of children with certain types of pediatric brain tumors; for example, low-grade gliomas now experience a five-year survival exceeding 75%. However, the prognosis for other types, such as high-grade gliomas, is still poor, and brain tumors remain the leading cause of childhood cancer-related deaths (1). In the setting of tumor recurrence or progression, the patient prognosis is substantially worse (2). Previous studies have reported distinct molecular patterns between initial and recurrent or progressive pediatric brain tumors of the same individual (3, 4, 5). In one study by Morrissy *et al*.(4), whole-genome sequencing of 33 pairs of human diagnostic and post-therapy medulloblastomas demonstrated substantial genetic divergence of the dominant clone after therapy. Petralia *et al*. (3) examined proteomic and genomic profiles of 18 pairs of surgical samples of pediatric brain tumors from two distinct disease occurrences of the same individuals, whereby the recurrent or progressive tumors appeared largely distinct from their initial tumor counterparts. In our recent survey of somatic Structural Variants (SVs) in pediatric brain tumors (5), increased numbers of SVs involved recurrent or progressive tumors as compared to the initial tumor from the same patient, with a set of 34 genes identified as having SV-mediated altered expression specifically involving recurrent or progressive tumors.

Our present study aimed to identify molecular changes in recurrent or progressive pediatric brain tumors compared to the corresponding initial tumors from the same patients. We analyzed genomic, transcriptomic, and proteomic data from a unique and large cohort of patients from The Children's Brain Tumor Tissue Consortium (CBTTC). The CBTTC has generated open-access genomics data on pediatric brain tumors and has provided these resources to the research community (6). Whereas pediatric brain tumors represent a collection of diseases, each defined by distinct histologic and molecular features, CBTTC data offer analysis opportunities to identify molecular patterns cutting across the many histologic types represented in its cohort. Importantly, a subset of CBTTC tumors represents multiple tumors taken from the same patient, including paired recurrent or progressive tumors along with the initial tumor. While we expect many individual instances of molecular differences between recurrent or progressive and initial tumors for any given patient, the availability of molecular data from large numbers of patients allows us to identify consistent changes across multiple patients as well as spanning diverse tumor histologic types. The set of genes showing consistent differences may provide molecular clues about the processes or pathways underlying more aggressive pediatric brain tumors.

## Materials and Methods

### Patient cohorts

Results are based upon data generated by the Children's Brain Tumor Network (CBTN). Specifically, we analyzed data from patients in the CBTTC cohort of the CBTN. We carried out RNA-sequencing (RNA-seq) analysis (at 30x coverage) for 63 recurrent or progressive tumors from 55 patients for which the corresponding initial tumor was also profiled (n=118 tumors in all). Of the tumors with RNA-seq data, 96 tumors from 44 unique patients were profiled (paired tumor and normal) by whole-genome sequence (WGS) analysis (at 60x coverage, 30X for germline), where each patient had an initial tumor and at least one recurrent or progressive tumor with data by WGS. Of the tumors with RNA-seq data, 30 tumors from 15 unique patients were profiled for proteomic expression by mass spectrometry, where each patient had an initial tumor and one recurrent or progressive tumor profiled. Of the 18 tumor pairs analyzed in the Clinical Proteomic Tumor Analysis Consortium (CPTAC) study of CBTTC proteomic data for initial versus recurrent/progressive comparisons (3), five did not involve an initial tumor, and the CPTAC data portal provided data on two additional initial tumor-progressive tumor pairs. In addition, RNA-seq data for another set of 806 CBTTC tumors from 806 patients (not included in the above 55 patients and 118 tumors), which patients did not have paired initial and recurrent or progressive tumors, were scored for a gene signature obtained from the paired recurrent/progressive versus initial tumor expression analysis.

Tumor samples in CBTTC spanned at least 33 different tumor histologic types, the most represented types including the following: ATRT, Atypical Teratoid Rhabdoid Tumor; CHDM, Chordoma; CPP, Choroid plexus papilloma; CRANIO, Craniopharyngioma; DIPG, Diffuse intrinsic pontine glioma; DNT, Dysembryoplastic neuroepithelial tumor; EPMT, Ependymoma; ES, Ewing's Sarcoma; GMN, Germinoma; GNG, Ganglioglioma; GNOS, Glial-neuronal tumor not otherwise specified (NOS); MBL, Medulloblastoma; MNG, Meningioma; NFIB, Neurofibroma/Plexiform; PBL, Pineoblastoma; PHGG, High-grade glioma/astrocytoma (WHO grade III/IV); PLGG, Low-grade glioma/astrocytoma (WHO grade I/II); PNET, Supratentorial or Spinal Cord primitive neuroectodermal; SCHW, Schwannoma; TT, Teratoma. As provided by the individual CBTN member institutions contributing the samples, the histologic designations of the tumors were confirmed by independent pathology review at the CBTN centralized biorepository, with most contributing sites providing representative histology slides. Samples were collected at the time of surgery or autopsy, flash-frozen, and stored in the Biorepository Resource Center at Children's Hospital of Philadelphia. The contributing sites provided clinical annotation, including tumor status (initial, recurrent, or progressive).

### Molecular profiling datasets

Tumor molecular profiling data were generated through informed consent as part of CBTN efforts and analyzed here per CBTN's data use guidelines and restrictions. We obtained processed RNA-seq data for CBTTC tumors from the PedCBioPortal (https://pedcbioportal.org/). RNA-seq data were quantile normalized before the analyses.

We accessed the somatic Structural Variant (SV) calls (Manta v1.4.0 algorithm) through the public project on Cavatica at https://cavatica.sbgenomics.com/u/cavatica/pbta-cbttc/files/#q?path=structural-variations. CBTTC used both Strelka2 v2.9.3 and Mutect2 v4.1.10 to call simple variants, i.e., single nucleotide variants (SNV) and insertions/deletions (INDEL), based on WGS data. We assessed the somatic variant MAFs through the public project on Cavatica at https://cavatica.sbgenomics.com/u/cavatica/pbta-cbttc/files/#q?path=simple-variants. Variant calls passing quality filters made by either Strelka2 or Mutect2 were considered, as the focus of this study was on SNVs and indels involving already known cancer genes (7) and hotspot residues (8), and with allowances made for the lower sequencing coverage of WGS as compared to that of whole-exome sequencing (WXS). Gene-level copy number alteration (CNA) calls, made based on CBTTC WGS data, were obtained from the PedCBioPortal (https://pedcbioportal.org/datasets). High-level gene amplification (approximating five or more copies) or deep copy loss (approximating gene deletion) were based on the “thresholded” calls (with values of +2 or -2, respectively) as made available by PedCBioPortal.

CPTAC, as part of its Pediatric Brain Cancer Pilot Study (3), generated the mass spectrometry-based proteomics data used in this publication. Samples were analyzed using the 11-plexed isobaric tandem mass tags (TMT-11). We obtained processed protein expression data from the CPTAC Data Portal (https://cptac-data-portal.georgetown.edu/cptacPublic/) (9). Taking the expression values provided in the Protein Report provided by CPTAC Data Portal, we normalized each CPTAC proteomic dataset for downstream analyses as done previously (10): first, log2 expression values were normalized to standard deviations from the median within each proteomic profile; next, expression values were normalized across samples to standard deviations from the median.

### Analysis of somatic variants

Somatic DNA events for a set of cancer-associated genes (5, 11) were examined across 96 tumors from 44 patients for which CBTTC profiled, by WGS, both an initial tumor and at least one recurrent or progressive tumor. For SNV and indels, we focused on a manually curated set of genes involving key pathways (5). For known oncogenes, if an SNV occurred in “hotspot” residues by Chang *et al*.(8), the SNV was included in the analysis. For putative tumor suppressor genes, we included all hotspot SNVs, inactivating SNVs (nonstop/nonsense), and indels. For CNA events, we focused on genes with cancer association by COSMIC (11) and either deep deletion or high-level amplification. For somatic SV events, we focused on COSMIC genes with a significant SV-expression association (p<0.01) for the 1MB region (incorporating tumor type and CNA) across the entire CBTTC cohort of 854 tumors by previous analyses (5). We defined SV-associated altered expression in a given tumor as having a breakpoint falling within 1Mb of the given gene, with expression>0.4SD or <-0.4SD from the median for the given tumor for genes with a global positive or negative association, respectively, between SV and expression (with the median defined above using the entire 854 CBTTC WGS cohort). Using the above, we tabulated all somatic events detected in a recurrent or progressive tumor but not in the initial tumor from the same patient.

### Differential expression analyses

Taking the CBTTC RNA-seq data for 63 recurrent or progressive tumors from 55 patients for which the corresponding initial tumor was also profiled, a paired test between recurrent/progressive and initial tumor groups (using log2-transformed values) was carried out for each gene using limma method (12). In all, we tested 16503 genes (median>0 for the 118 tumors) in the paired analysis. The expected numbers of genes that would be nominally significant due to multiple testing, based on probability, were also considered. Taking the CBTTC/CPTAC proteomic dataset for 15 recurrent or progressive tumors from 15 patients with the corresponding initial tumor also profiled, we carried out a paired t-test between recurrent/progressive and initial tumor groups (using normalized values) for each protein. The paired analysis served to control differences between histologic types, as we evaluated relative differences between each recurrent or progressive tumor and its paired initial tumor reference across the dataset. We evaluated the overall concordance between the CBTTC mRNA results and protein results using Gene Set Enrichment Analysis (GSEA) method (13). The GSEA ranked gene list was based on differential protein expression using paired t-statistic (based on 7154 genes with data). The sets of genes found nominally significant in the paired analysis may have relatively high expected false positive rates (14). We therefore carried out integration of the differential patterns observed in the CBTTC cohort by RNA-seq platform with other platforms or with other patient cohorts, following a similar approach as that of the GSEA method, which aims to identify significant enrichment patterns even in instances where the differential gene sets involved do not have low false discovery rates (15).

We applied the ESTIMATE algorithm (16) to the CBTTC RNA-seq dataset, which algorithm estimates relative tumor sample purity based on gene expression patterns. To determine whether the differential expression patterns associated with recurrent or progressive tumors might be attributable entirely to tumor sample purity differences, we carried out a regression analysis with the top 68 genes with differential expression with p<0.001 by limma, whereby ESTIMATE scores were included as a covariate for the association of each gene with tumor status. In this regression analysis, the paired initial tumors were included but with zero values for both gene expression and ESTIMATE score, while the recurrent or progressive tumors had values centered on the corresponding initial tumor pair. All 68 genes significant with p<0.001 (by limma) were also significant with p<0.01 by regression model incorporating ESTIMATE score, meaning that the ESTIMATE score alone could not explain away the significant differences observed for each gene. Neither the CPTAC nor the TCGA paired samples showed any significant differences in estimated tumor sample purities between recurrent/progressive versus initial tumors by ESTIMATE.

### Survival analyses

We scored RNA-seq data for an independent set of 806 CBTTC tumors from 806 patients (not included in the 55 patients with paired initial and recurrent/progressive tumors) or a gene signature obtained from the paired recurrent/progressive versus initial tumor expression analysis (based on p<0.001 by paired limma test). We centered log2 gene expression values on the median across samples. The centered expression profiles were each scored for the recurrent/progressive signature using our t-score metric (17), which metric is high when the genes found high or low in recurrent/progressive tumors also appear relatively high or low, respectively, in the external profile. We evaluated the t-score as a continuous variable for association with patient survival using the Cox model. We also binned patients into thirds based on the tumor t-score and evaluated differences in survival using a log-rank test. In addition, we used stratified Cox models or stratified log-rank to evaluate survival association when correcting for tumor histologic type. With the stratified analysis, the p-value for the association with worse outcome is significant only if histology alone cannot explain the survival differences observed.

### Comparisons with adult glioma results

For The Cancer Genome Atlas (TCGA) glioma cases (GBM and LGG projects), we obtained RNA-seq data from The Broad Institute Firehose pipeline (http://gdac.broadinstitute.org/). All RNA-seq sample profiles were aligned using the by UNC RNA-seq V2 pipeline (17). Recurrent and paired initial tumors from 20 adult glioma patients from TCGA were examined for differential expression by paired limma applied to each gene. We evaluated the overall concordance between the CBTTC results and TCGA results using GSEA (13). The GSEA ranked gene list was based on differential mRNA expression in TCGA glioma using paired limma t-statistic (based on 16152 genes represented in both datasets).

### Unsupervised clustering

ConsensusClusterPlus R-package (18) was used to identify the structure and relationship of the CBTTC progressive or recurrent tumors, as normalized to their initial tumor pair. Taking the RNA-seq dataset of 63 recurrent or progressive tumors from 55 patients for which the corresponding initial tumor was also profiled, we centered the log2 expression values on the value of the initial tumor. We selected the top 2000 most variable mRNAs from the centered dataset, according to standard deviation, for unsupervised clustering analysis. Consensus ward linkage hierarchical clustering identified k=2 to k=10 subtypes, with the stability of the clustering increasing with increasing k. Taking the k=5 cluster solution, we defined for each tumor subset the top 200 genes highest in the given subset versus the rest of the differential tumor profiles (using unpaired t-test of the centered expression values). The unsupervised discovery involved differential expression profiles of each recurrent or progressive tumor subtracted from its corresponding initial tumor pair, which essentially corrected for histologic differences. Therefore, the sample profiles did not segregate according to tumor histology in this analysis, which would be the case had the clustering analysis been carried out on the original dataset (5).

### Statistics

All P-values were two-sided unless otherwise specified. We assessed differential expression using paired limma or paired t-test. One-sided Fisher's exact tests determined the significance of overlap between two given feature lists. We carried out GSEA (13) using version 4.0.3 of the software, with GSEAPreranked feature and classic enrichment statistic. We evaluated Gene Ontology (GO) annotation term enrichment within sets of differentially expressed genes using SigTerms software (19) and one-sided Fisher's exact tests. Visualization by heat maps used JavaTreeview (20) and matrix2png (version 1.2.1) (21).

### Ethics

Tumor molecular profiling data were generated through informed consent involving institutional review boards as part of CBTTC efforts and analyzed here per CBTTC's data use guidelines and restrictions.

### Role of funders

The funder had no role in study design, data collection, data analysis, data interpretation, or writing of the report. The corresponding authors had full access to all the data in the study and had final responsibility for the decision to submit for publication.

## Results

### DNA-level alterations

Our study focused on RNA-seq data for 118 pediatric brain tumor samples from the CBTTC, representing 55 patients with both an initial tumor (n=55) and one or more recurrent or progressive tumors (n=63). Of the tumors with RNA-seq data, 96 tumors from 44 unique patients were profiled by WGS, with each patient having both an initial tumor and one or more recurrent or progressive tumors. Tumor samples in the 118-patient cohort spanned 16 different tumor types based on histology (Data File S1), the more represented types including: low-grade glioma/astrocytoma (n=23 tumors), high-grade glioma/astrocytoma (n=18), medulloblastoma (n=16), ependymoma (n=16), atypical teratoid rhabdoid tumor (n=8), and meningioma (n=7). Our molecular analyses below focused on paired comparisons between a patient's recurrent or progressive tumor and the corresponding initial tumor, which served to control for inherent differences between histologic types.

**Figure 1:**
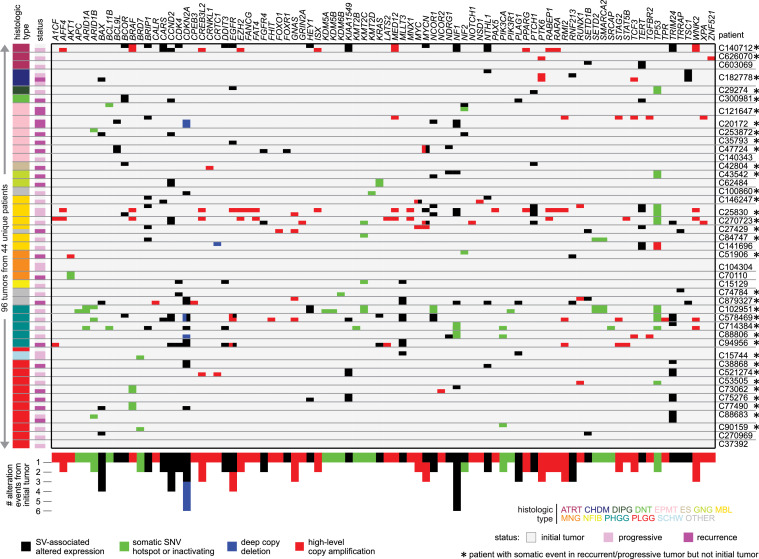
DNA alterations involving recurrence or progression of pediatric brain tumors. Somatic DNA events for a set of cancer-associated genes (5, 11) are represented across 96 tumors from 44 patients for which CBTTC profiled, by WGS, both an initial tumor and at least one recurrent or progressive tumor. Tumor status color bar denotes initial tumor, progressive, or recurrence. Tumors are organized by histology of the initial tumor followed by the tumor pair (the initial tumor being represented first for each patient). Black matrix entry represents SV breakpoint in proximity to the gene (within 1 Mb of the gene start) and associated with altered gene expression (> 0.4SD or < −0.4SD from the median of the CBTTC WGS cohort (5) for the case harboring the breakpoint). Green represents somatic SNV/indel (either missense SNV within hotspot residue (8) or inactivating mutation by indel/nonsense/nonstop, with only hotspot mutations being considered for oncogenes). Red or blue represents high-level amplification or deep deletion, respectively. As tabulated in the bar chart along the bottom, “alteration events from initial tumor” are somatic events detected in a recurrent or progressive tumor but not in the initial tumor from the same patient. Of the 44 patients represented here, 35 involve at least one gene with an alteration event in a recurrent or progressive tumor that was not present in the initial tumor.

By WGS, we examined somatic DNA events for a set of cancer-associated genes (5, 11) (including COSMIC (11) genes and genes involved in key oncogenic or tumor-suppressive pathways (5), see Methods), in recurrent or progressive tumors having a paired initial tumor from the same patient (Figure 1 and Data File S2). Interestingly, increased overall numbers of somatic SNVs or indels were detected on average in recurrent or progressive tumors from a given patient, as compared to the initial tumor from the same patient (p=0.006, paired t-test). For selected genes, we considered somatic SNVs or indels, high-level amplification or deep deletion, and altered expression associated with nearby somatic SV breakpoints (which involve gene fusions such as *KIAA1549*-*BRAF* as well as altered gene cis-regulation (5)). Of the 44 patients examined, 35 involved at least one cancer-associated gene with an alteration event in a recurrent or progressive tumor that was not present in the initial tumor, involving 86 cancer-associated genes. On average, the 44 patients had 3.15 events involving somatic gene alteration in a recurrent or progressive tumor but not in the initial tumor. Genes most frequently involved in the above events included *NF1* (n=6 events, SV-associated), *CDKN2A* (n=6, SV or copy loss), *BAX* (n=4, SV), *CCND2* (n=4, SV), *EGFR* (n=4, SV or amplification), *CREB3L2* (n=3, amplification), *GNAS* (n=3, amplification), *MYCN* (n=3, SV or amplification), *PLAG1* (n=3, SV), *PTK6* (n=3, amplification), *RIM2* (n=3, amplification), *RNF213* (n=3, SV), and *WNK2* (n=3, amplification). In contrast, we also found events involving a somatic gene alteration found in the initial tumor but not in a corresponding recurrent or progressive tumor. Still, these latter events were statistically less common than the former (average of 1.52 versus 3.15 events across the 44 patients, p=0.005 by paired t-test).

### Altered gene and protein expression

**Figure 2:**
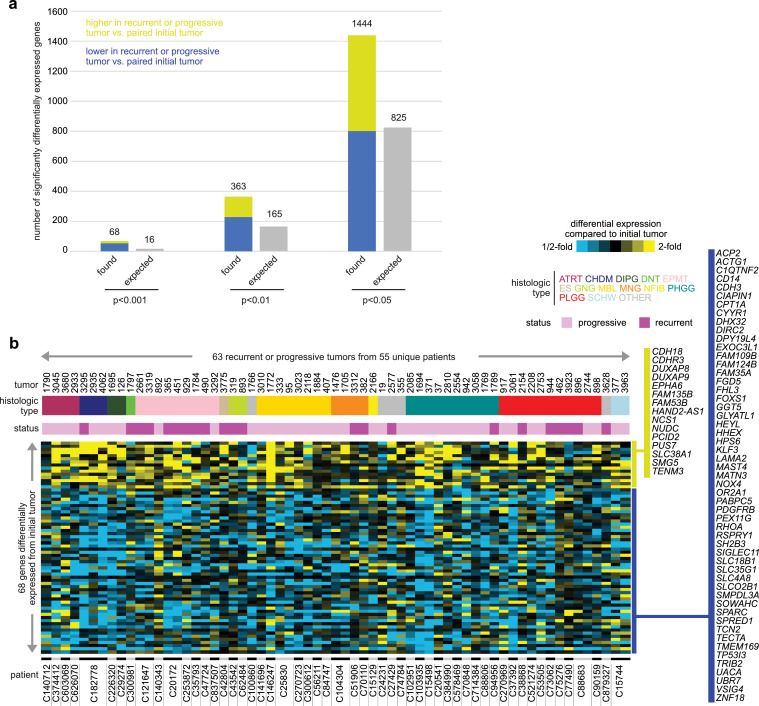
Altered gene transcription involving recurrence or progression of pediatric brain tumors. (a) Based on RNA-seq data, numbers of significantly differentially expressed genes (using p<0.001, p<0.01, and p<0.05 significance thresholds, respectively), comparing recurrent or progressive tumors with the patient's initial tumor. Analyses involve 63 recurrent or progressive tumors from 55 patients for which the corresponding initial tumor was also profiled. P-values by limma moderated paired t-test (12) using log2-transformed gene expression values. The numbers of genes expected by chance due to multiple testing involving 16504 genes analyzed are also represented. (b) Heat map of 68 genes differentially expressed (p<0.001, limma paired t-test) in recurrent or progressive tumors compared to the initial tumor. Gene values for each recurrent or progressive tumor represented are centered on the corresponding initial tumor. Initial tumors are not represented here, as these are the reference for each patient and would therefore appear as all black in a heat map representation.

Using RNA-seq data representing 16503 genes and 118 tumors from 55 patients (63 recurrent or progressive tumors and 55 paired initial tumors), we carried out a paired analysis for each gene between recurrent/progressive and initial tumor groups. At different significance thresholds, the numbers of significant genes exceeded the chance expected due to multiple testing of genes (Figure 2a and Data File S3). At p<0.001 (paired limma test), 68 mRNA transcripts (15 higher in recurrent/progressive, 53 lower) were significantly differentially expressed (Figure 2b), where we expected just ∼16 of these genes by chance. More nominally significant genes could be defined at more relaxed statistical cutoffs, though with a greater proportion of expected false positives. At p<0.01 or p<0.05, about twice as many significant genes over chance expected were found (Figure 2a). Despite sets of nominally significant genes having a relatively high expected false positive rate, the entire set would be enriched for true positives representing molecular information. Such information would represent real biological differences, which downstream analyses and integration may reveal with other datasets, as described below. For example, genes differentially expressed in recurrent/progressive tumors at p<0.01 were significantly enriched for gene categories by GO (Data Files S3), including ‘neurotransmitter transport’ (7 genes, p<1E-5, one-sided Fisher's exact test) and ‘anterograde trans-synaptic signaling’ (10 genes, p=0.0001) for the higher genes and ‘collagen-containing extracellular matrix’ (19 genes, p<1E-6) and ‘extracellular region’ (38 genes, p=0.00002) for the lower genes. We found somewhat higher estimated tumor sample purity on average in recurrent or progressive tumors versus initial tumors (p=0.03 paired t-test), while regression analyses indicated that this purity association would not explain the widespread differential expression patterns observed (see Methods).

**Figure 3:**
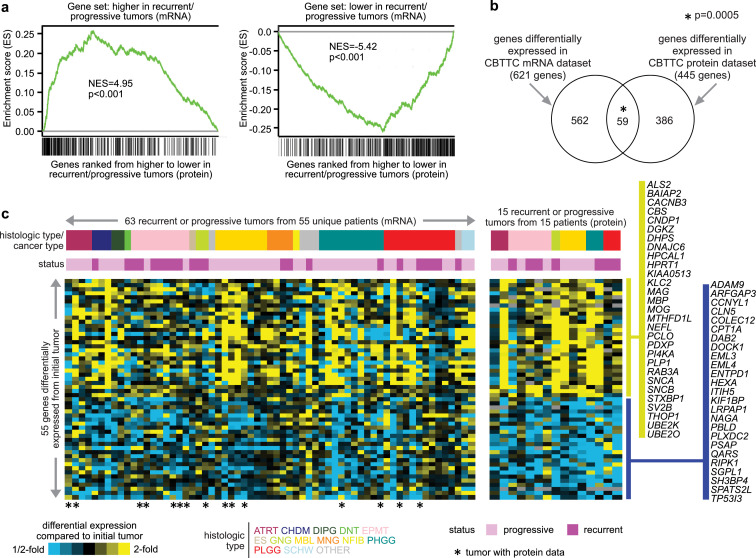
Altered protein expression involving recurrence or progression of pediatric brain tumors. (a) Recurrent or progressive and paired initial tumors from 15 pediatric brain tumor patients from CBTTC and CPTAC (3) were examined for differential protein expression by mass spectrometry. Profiled genes were ranked from higher to lower differential protein expression in recurrent or progressive versus initial tumors (based on 7154 genes with data), and the relative positions of genes with mRNAs higher or lower (two-sided p<0.05, limma paired t-test) in CBTTC pediatric recurrent or progressive tumors were evaluated for enrichment within the proteomic recurrent/progressive-associated patterns by GSEA method (13). Bar graphs represent normalized enrichment scores (NES) with associated significance of enrichment or anti-enrichment, respectively. (b) Venn diagram representing the overlap between the genes with differential protein expression and the genes with differential mRNA expression (p<0.05 for each) in recurrent/progressive tumors. P-value by one-sided Fisher's exact test. (c) Heat maps represent the 55 genes (from part b) having differential expression in recurrent or progressive tumors compared to the initial tumor, in the same direction for both mRNA and proteomic datasets. Gene values for each recurrent or progressive tumor represented are centered on the corresponding initial tumor.

Of the above 118 tumors with RNA-seq data, 30 tumors from 15 unique patients were profiled for proteomic expression by mass spectrometry (3), with an initial tumor and one recurrent or progressive tumor profiled for each patient. Out of 7154 genes with protein data, 445 were differentially expressed at the protein level with p<0.05 (paired t-test) between recurrent/progressive and initial tumor groups, which somewhat exceeded chance expectation (∼357 genes). At the same time, by GSEA (13), the differential proteomic patterns associated with recurrent or progressive tumors were broadly enriched, with consistent direction of change, for mRNAs higher or lower in recurrent or progressive tumors based on RNA-seq (Figure 3a). Between the 621 genes differentially expressed (p<0.05) at the mRNA level (based on the analysis of 118 tumors) and the 445 genes differentially expressed (p<0.05) at the protein level, 59 genes overlapped (Figure 3b), a significant number (p=0.0005, one-sided Fisher's exact test). Of the 59 genes, 55 has the same direction of change in both protein and mRNA data (Figure 3c), including genes with GO annotation ‘synaptic signaling’ (eight genes: *ALS2, CACNB3, MBP, PLP1, RAB3A, SNCB, STXBP1, SV2B*) among those higher in recurrent or progressive tumors, and ‘sphingolipid metabolic process’ genes (four genes: *HEXA, NAGA, PSAP, SGPL1*) among those lower in recurrent or progressive tumors.

To facilitate access to CBTTC transcriptomic and proteomic results by the general research community, we integrated CBTTC data with the UALCAN data portal (22), allowing users to query genes of interest for associations with recurrence/progression, tumor histology, or patient characteristics (http://ualcan.path.uab.edu/).

### Gene signature predicting patient outcome

**Figure 4:**
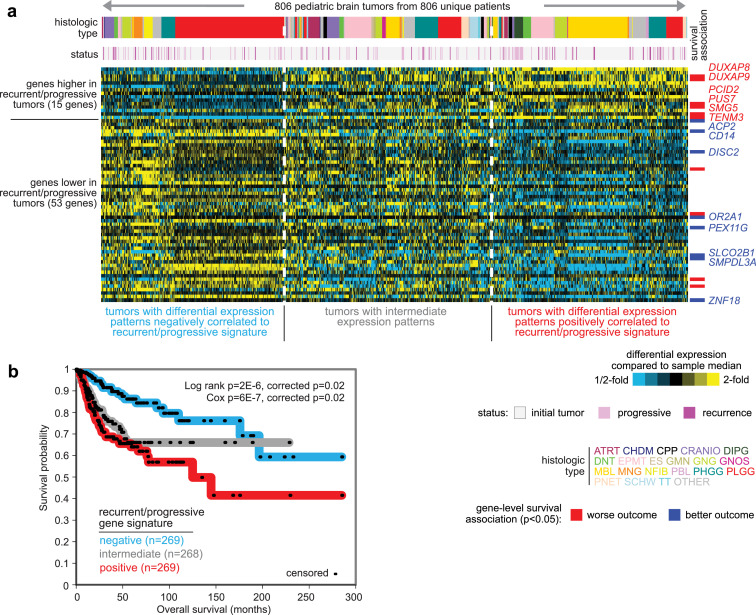
A gene transcription signature of recurrence or progression predicts patient outcome in an independent set of pediatric brain tumors. (a) RNA-seq data for the set of 806 CBTTC tumors from 806 patients (not included in the 55 patients with paired initial and recurrent/progressive tumors) were scored for the 68-gene signature from Figure 2b (see Methods). As shown in the heat map, the 806 tumors could be stratified into those with differential expression patterns either positively or negatively correlated to the recurrent/progressive signature and those with intermediate patterns not strongly correlated with the signature (grouping tumors into top third, middle third, and bottom third of signature scores). Genes individually associated with patient outcome in the 806-patient cohort (p<0.05, stratified Cox correcting for histologic type) and consistent with the signature are listed off to the right. (b) Kaplan-Meier plot represents the 68-gene recurrent/progressive transcriptional signature associating with overall survival in the 806-patient pediatric brain tumor set. P-values by log-rank test and by univariate Cox, as indicated. “Corrected” p-values correct for histologic type.

We hypothesized that the differential expression patterns between recurrent or progressive and initial tumors would represent molecular differences between more aggressive versus less aggressive cancers, respectively. To test this, we examined RNA-seq data for an independent set of 806 CBTTC pediatric brain tumor tumors from 806 patients, which patients did not have an initial tumor and recurrent or progressive pair (involving 667 initial, 40 recurrent, and 97 progressive tumors). Taking the above 68-gene signature from the paired recurrent/progressive versus initial tumor analysis (p<0.001, limma), we scored the 806 tumors based on the signature pattern (Figure 4a). Tumors with high signature scores showed relatively high or low expression for the genes higher or lower, respectively, in recurrent or progressive tumors. High signature scores were associated with worse overall survival (Figure 4b, p=6E-7, univariate Cox, p=0.02, stratified Cox correcting for tumor histologic type). Of the 68 genes, 14 individually associated with patient outcome in the 806-patient cohort (p<0.05, stratified Cox correcting for histologic type) and consistent with the signature direction of change: *DUXAP8, DUXAP9, PCID2, PUS7, SMG7*, and *TENM3*, associated with worse overall survival; *ACP2, CD14, DISC2, OR2A1, PEX11G, SLCO2B1, SMPDL3A*, and *ZNF18*, associated with better overall survival. Estimated tumor sample purities were higher on average in the poor prognosis group as compared to the other two groups (p=1E-50, t-test), where molecular subtypes in general frequently involve differences according to sample purity, which may entail the involvement of the tumor microenvironment (7, 23).

### Comparison with altered expression in adult gliomas

**Figure 5:**
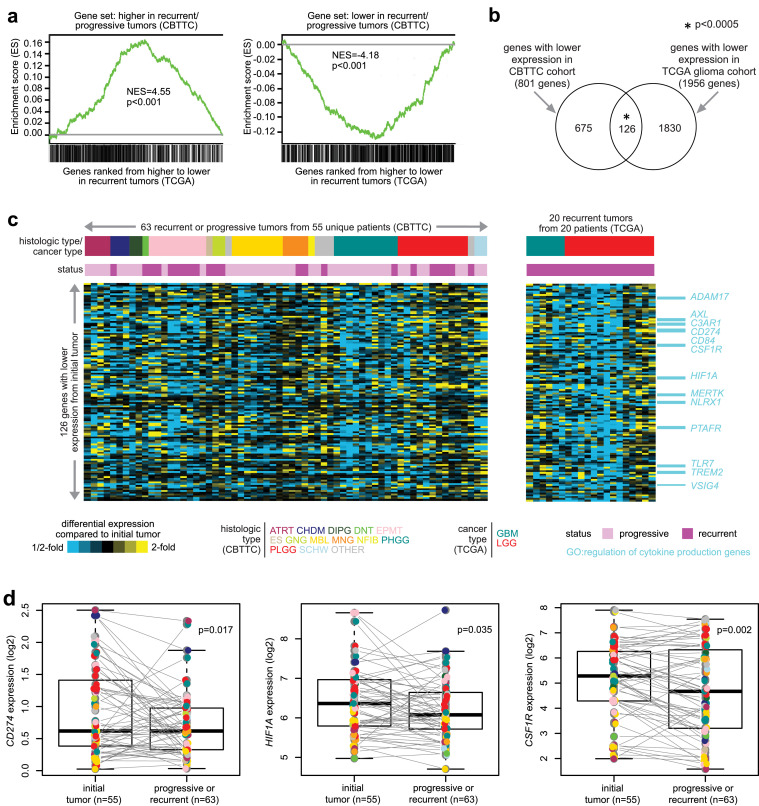
Overlapping gene transcription signatures of recurrence or progression between pediatric brain tumors and adult gliomas. (a) Recurrent and paired initial tumors from 20 adult glioma patients from TCGA (25) were examined for differential expression by RNA-seq. Profiled genes were ranked from higher to lower differential expression in TCGA recurrent versus initial tumors, and the relative positions of genes higher or lower (two-sided p<0.05, limma paired t-test) in CBTTC pediatric recurrent or progressive tumors were evaluated for enrichment within TCGA recurrent-associated patterns by GSEA method (13). Bar graphs represent normalized enrichment scores (NES) with the associated significance of enrichment or anti-enrichment, respectively. (b) Venn diagram representing the overlap between the genes with lower expression in recurrent/progressive tumors in CBTTC cohort (p<0.05, two-sided) and the genes with lower expression in recurrent tumors in TCGA cohort (p<0.05, one-sided). P-value by one-sided Fisher's exact test. (c) Heat maps represent the 126 genes (from part b) having lower expression in recurrent or progressive tumors compared to the initial tumor, in both CBTTC pediatric and TCGA adult glioma cohorts. Gene values for each recurrent or progressive tumor represented are centered on the corresponding initial tumor. Genes with GO annotation ‘regulation of cytokine production genes’ are listed individually off to the right. (d) In CBTTC cohort, box plots representing differential expression between recurrent or progressive tumors and paired initial tumors for selected genes *CD274, HIF1A*, and *CSF1R*. P-values by limma moderated paired t-test using log2-transformed gene expression values. Box plot represents 5% (lower whisker), 25% (lower box), 50% (median), 75% (upper box), and 95% (upper whisker). Data points are colored according to tumor histologic type, using the color scheme in part c. Lines are drawn between tumor pairs, with most pairs showing decrease in the progressive or recurrent tumor for each of these genes.

Notwithstanding the extensive molecular differences between pediatric and adult brain tumors (5, 24), we hypothesized that a portion of the genes differentially expressed between recurrent or progressive pediatric brain tumors and initial tumors might also be involved in recurrent brain tumors from adult patients. We examined RNA-seq data from recurrent and paired initial tumors from 20 adult glioma patients from TCGA (25). By GSEA (13), the differential mRNA patterns associated with adult recurrent gliomas were broadly enriched, with consistent direction of change, for mRNAs higher or lower in recurrent or progressive pediatric brain tumors (Figure 5a). In particular, between the 801 genes with lower expression in pediatric recurrent or progressive tumors (p<0.05, paired limma, two-sided) and the 1956 genes with lower expression in adult recurrent tumors (p<0.05, paired limma, one-sided), 126 genes overlapped (Figures 5b and 5c), a significant number (p<0.0005, one-sided Fisher's exact test). On the other hand, the genes higher in recurrent or progressive tumors did not significantly overlap between pediatric and adult tumors (51 genes, p=0.34, one-sided Fisher's exact test). The 126 shared genes lower in both pediatric and adult tumors were enriched (p=0.0003, one-sided Fisher's exact test) for genes with GO annotation ‘regulation of cytokine production genes’, including *CD274, CSF1R*, and *HIF1A* (Figures 5c and 5d).

### Altered gene expression within specific tumor subsets

**Figure 6:**
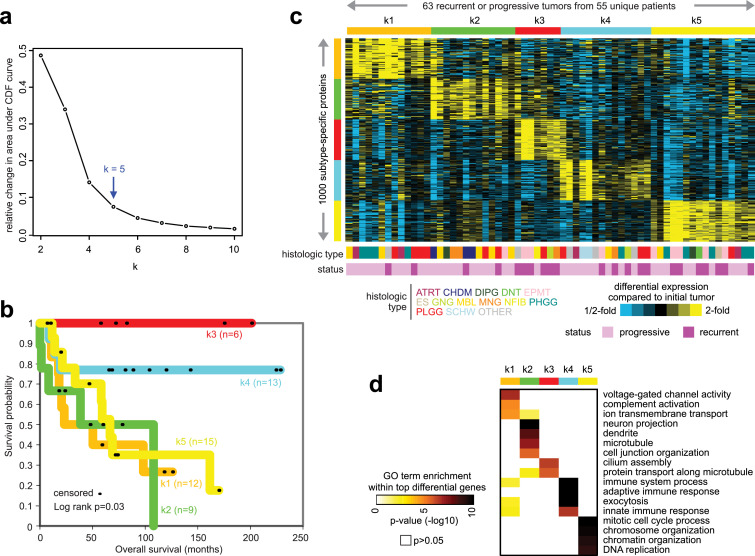
Altered gene transcription involving recurrence or progression within specific subsets of pediatric brain tumors. (a) Taking the differential expression profiles for the 63 recurrent or progressive tumors (each profile centered on the corresponding initial tumor), ConsensusClusterPlus (18) clustering algorithm was applied to identify tumor subsets. Delta area plot graphic shows the relative change in area under the cumulative distribution function (CDF) curve comparing k and k − 1. For k = 2, there is no k -1, so the total area under the curve rather than the relative increase is plotted. This graphic allows one to determine the relative increase in consensus and determine k at which there is no appreciable increase. (b) Differences in patient overall survival among the five tumor subsets within the cohort of 55 patients with paired recurrent or progressive tumor. P values by log-rank test, evaluating for significant differences among the groups. (c) Differential expression patterns for a set of 1000 genes help distinguish between the five tumor subsets from part a (for each subset, showing the top 200 genes highest in the given subset versus the rest of the differential tumor profiles). (d) For the top over-expressed genes associated with each tumor subset (from part c), represented categories by GO were assessed, with selected enriched categories represented here. P-values by one-sided Fisher's exact test.

Where the above expression analyses could identify genes with statistically consistent changes across the 63 recurrent or progressive tumors examined, we hypothesized that some genes are changed in only a subset of tumors. We carried out unsupervised clustering analysis of the differential expression profiles of recurrent or progressive tumors to identify such patterns. Centering the 63 recurrent or progressive pediatric brain tumor RNA-seq profiles on the corresponding initial tumors, we selected the top 2000 most variable genes and defined five subsets of recurrent or progressive tumors (Figure 6a), each subset consisting of seven to 16 tumors of diverse histologic types. Of the seven patients with multiple recurrent or progressive tumors (eight tumors in all), only one had different tumors falling within different subsets. We observed significant differences in overall patient survival among the five subsets (Figure 6b, p=0.03, log-rank test), reflecting true biological differences among them, with ‘k3’ (n=6 patients) and ‘k4’ (n=13) subsets associated with markedly better outcome compared to the other subsets. For each recurrent/progressive tumor subset, we identified the top genes most highly expressed in the given subset but not across the rest of the tumors (Figure 6c), which again showed the tumor subsets to be distinct from each other. Within the top differentially expressed genes underscoring each tumor subset, specific GO gene categories were over-represented (Figure 6d and Data File S4). The ‘k3’ and ‘k4’ tumors involving better survival were respectively associated with GO terms ‘cilium assembly’ and ‘immune system process’. Of the tumor subsets involving worse patient outcome, ‘k1’ tumors involved GO terms ‘voltage-gated channel activity’ and ‘complement activation’; ‘k2’ tumors involved GO terms ‘neuron projection’, ‘dendrite’, and ‘microtubule’; and ‘k5’ tumors involved GO terms 'mitotic cell cycle process' and 'DNA replication'. Estimated tumor sample purity levels were higher in the k5 subtype as compared to the other subtypes (ANOVA p=0.003; p=0.0004, t-test). The above findings would suggest multiple and distinct pathways being involved with advanced disease within different tumor subsets, in addition to genes commonly altered on average across all tumors.

## Discussion

Here, we identified consistent gene changes between recurrent or progressive pediatric brain tumors compared to the corresponding initial tumor across multiple patients. While genomic or transcriptomic changes in a recurrent or progressive tumor may be specific to a given patient, molecular changes that would be common across multiple patients and diverse histologic types may point to pathways or processes underlying more aggressive disease. At the DNA level, we observed changes within individual patients, but no consistent changes observed across all samples or all samples of a given histology. In contrast, At the RNA or protein level, we observed both consistent changes across all samples and very robust changes within subsets of patients. The multiple patient tumor samplings and large sample sizes represented by the CBTTC datasets provided the power needed to identify significant differences over chance expected. Future studies utilizing even greater patient numbers could help further refine the gene expression signatures of recurrence or progression. The sets of nominally significant genes identified in this present study would contain a certain level of statistical noise. Also, not all differentially expressed genes observed may play an important role in the disease. Therefore, an important aspect of our study was integrating the differential patterns observed in the CBTTC cohort by RNA-seq platform with other platforms or with other patient cohorts. Significant numbers of genes overlapped between transcriptomic and proteomic datasets and between pediatric and adult brain tumors. Such genes with consistent changes observed across multiple datasets would seem likely to represent true changes within pediatric brain tumors, even if the significance levels of these genes might be nominal for the CBTTC RNA-seq dataset.

Both the individual genes and their associated gene categories involved in differential expression in recurrent or progressive tumors could represent possible mediators of disease progression. In addition to genes showing consistent differences in expression across all tumors examined, unsupervised clustering analysis could identify genes with differential expression within specific subsets of recurrent or progressive tumors. For example, genes with increased expression on average across all recurrent or progressive tumors included ‘neurotransmitter transport’ genes, while, in the clustering analysis, genes high with one of the recurrent/progressive tumor subsets associated with worse outcomes included ‘neuron projection’ and ‘dendrite’ genes. Elsewhere, neuronal activity has been shown to promote the growth of a range of pediatric brain tumor types (26). Another CBTTC recurrent/progressive tumor subset involved better patient prognosis and high expression of adaptive and innate immune response genes. In another study, low-grade tumors have been characterized by greater T-cell density than high-grade pediatric gliomas, and a decline in tumor-infiltrating T-cells characterized recurrent tumors (27). Interestingly, genes involved with ‘mitotic cell cycle process’ and ‘DNA replication’ were highly expressed only within a subset of recurrent or progressive tumors, suggesting that other processes may be involved in most of the other tumors. With gene-level associations with recurrence or progression, our results would represent a resource for identifying gene candidates for further investigation. To extend the resource potential of CBTTC data, our user-friendly UALCAN data portal (22) facilitates differential analyses by individual gene or protein.

## Declaration of Competing Interest

The authors declare no competing interests.

## Appendix Supplementary materials

Image, application 1

Image, application 2

Image, application 3

Image, application 4

## References

[1] Curtin S., Minino A., Anderson R. (2016). Declines in cancer death rates among children and adolescents in the United States, 1999–2014. NCHS Data Brief.

[2] Pollack I. (1994). Brain tumors in children. N Engl J Med.

[3] Petralia F., Tignor N., Reva B., Koptyra M., Chowdhury S., Rykunov D. (2020). Integrated Proteogenomic Characterization across Major Histological Types of Pediatric Brain Cancer. Cell.

[4] Morrissy A., Garzia L., Shih D., Zuyderduyn S., Huang X., Skowron P. (2016). Divergent clonal selection dominates medulloblastoma at recurrence. Nature.

[5] Zhang Y., Chen F., Donehower L., Scheurer M., Creighton C. (2021). A pediatric brain tumor atlas of genes deregulated by somatic genomic rearrangement. Nat Commun.

[6] Ijaz H., Koptyra M., Gaonkar K., Rokita J., Baubet V., Tauhid L. (2019). Pediatric High Grade Glioma Resources from the Children's Brain Tumor Tissue Consortium (Cbttc). Neuro Oncol.

[7] Chen F., Zhang Y., Gibbons D., Deneen B., Kwiatkowski D., Ittmann M. (2018). Pan-cancer molecular classes transcending tumor lineage across 32 cancer types, multiple data platforms, and over 10,000 cases. Clin Cancer Res.

[8] Chang M., Asthana S., Gao S., Lee B., Chapman J., Kandoth C. (2016). Identifying recurrent mutations in cancer reveals widespread lineage diversity and mutational specificity. Nat Biotechnol.

[9] Edwards N., Oberti M., Thangudu R., Cai S., McGarvey P., Jacob S. (2015). The CPTAC Data Portal: A Resource for Cancer Proteomics Research. J Proteome Res.

[10] Monsivais D., Vasquez Y., Chen F., Zhang Y., Chandrashekar D., Faver J. (2021). Mass-spectrometry-based proteomic correlates of grade and stage reveal pathways and kinases associated with aggressive human cancers. Oncogene.

[11] Forbes S., Beare D., Boutselakis H., Bamford S., Bindal N., Tate J. (2017). COSMIC: somatic cancer genetics at high-resolution. Nucleic Acids Res.

[12] Ritchie M., Phipson B., Wu D., Hu Y., Law C., Shi W. (2015). limma powers differential expression analyses for RNA-sequencing and microarray studies. Nucleic Acids Res.

[13] Subramanian A., Tamayo P., Mootha V., Mukherjee S., Ebert B., Gillette M. (2005). Gene set enrichment analysis: a knowledge-based approach for interpreting genome-wide expression profiles. Proc Natl Acad Sci U S A..

[14] Storey J.D., Tibshirani R. (2003). Statistical significance for genomewide studies. Proc Natl Acad Sci USA.

[15] Mootha V.K., Lindgren C.M., Eriksson K.F., Subramanian A., Sihag S., Lehar J. (2003). PGC-1alpha-responsive genes involved in oxidative phosphorylation are coordinately downregulated in human diabetes. Nature genetics.

[16] Yoshihara K., Shahmoradgoli M., Martínez E., Vegesna R., Kim H., Torres-Garcia W. (2013). Inferring tumour purity and stromal and immune cell admixture from expression data. Nat Commun.

[17] The_Cancer_Genome_Atlas_Research_Network (2013). Comprehensive molecular characterization of clear cell renal cell carcinoma. Nature.

[18] Wilkerson M., Hayes D. (2010). ConsensusClusterPlus: a class discovery tool with confidence assessments and item tracking. Bioinformatics.

[19] Creighton C., Nagaraja A., Hanash S., Matzuk M., Gunaratne P. (2008). A bioinformatics tool for linking gene expression profiling results with public databases of microRNA target predictions. RNA.

[20] Saldanha AJ. (2004). Java Treeview–extensible visualization of microarray data. Bioinformatics.

[21] Pavlidis P., Noble W. (2003). Matrix2png: A Utility for Visualizing Matrix Data. Bioinformatics.

[22] Chandrashekar D., Bashel B., Balasubramanya S., Creighton C., Ponce-Rodriguez I., Chakravarthi B. (2017). UALCAN: A Portal for Facilitating Tumor Subgroup Gene Expression and Survival Analyses. Neoplasia.

[23] Chen F., Chandrashekar D., Varambally S., Creighton C. (2019). Pan-cancer molecular subtypes revealed by mass-spectrometry-based proteomic characterization of more than 500 human cancers. Nat Commun.

[24] Glod J., Rahme G., Kaur H., H Raabe E., Hwang E., Israel M. (2016). Pediatric Brain Tumors: Current Knowledge and Therapeutic Opportunities. J Pediatr Hematol Oncol.

[25] Ceccarelli M., Barthel F., Malta T., Sabedot T., Salama S., Murray B. (2016). Molecular Profiling Reveals Biologically Discrete Subsets and Pathways of Progression in Diffuse Glioma. Cell.

[26] Venkatesh H., Johung T., Caretti V., Noll A., Tang Y., Nagaraja S. (2015). Neuronal Activity Promotes Glioma Growth through Neuroligin-3 Secretion. Cell.

[27] Robinson M., Vasquez J., Kaushal A., MacDonald T., Velázquez Vega J., Schniederjan M. (2020). Subtype and grade-dependent spatial heterogeneity of T-cell infiltration in pediatric glioma. J Immunother Cancer.

